# Assessment of immune organ dysfunction in critical illness: utility of innate immune response markers

**DOI:** 10.1186/s40635-017-0163-0

**Published:** 2017-10-23

**Authors:** Carmen Andrea Pfortmueller, Christian Meisel, Michaela Fux, Joerg C. Schefold

**Affiliations:** 1 Department of Intensive Care Medicine, Inselspital, Bern University Hospital, University of Bern, Freiburgstrasse 10, 3010 Bern, Switzerland; 2 Department of Medical Immunology, Charité University Hospital Berlin, Augustenburger Platz 1, 13353 Berlin, Germany; 3 Department of Immunology, Labor Berlin Charité Vivantes, Sylter Strasse 2, 13353 Berlin, Germany; 4 University Institute of Clinical Chemistry, Inselspital, Bern University Hospital, University of Bern, Bern, Switzerland

**Keywords:** Sepsis, Sepsis-associated immunosuppression, Biomarkers, Critical illness, Immune function, Immunomodulation, Immune modulation, Immune suppression, HLA-DR expression, mHLA-DR

## Abstract

In critically ill patients, organ dysfunctions are routinely assessed, monitored, and treated. Mounting data show that substantial critical illness-induced changes in the immune system can be observed in most ICU patients and that not only “hyper-inflammation” but also persistence of an anti-inflammatory phenotype (as in sepsis-associated immunosuppression) is associated with increased morbidity and mortality. Despite common perception, changes in functional immunity cannot be adequately assessed by routine inflammatory biomarkers such as C-reactive protein, procalcitonin, or numerical analysis of leukocyte (sub)-counts. Cytokines appear also not suited due to their short half-life and pleiotropy, their unexclusive origin from immune cells, and their potential to undergo antagonization by circulating inactivating molecules. Thus, beyond leukocyte quantification and use of routine biomarkers, direct assessment of immune cell *function* seems required to characterize the immune systems’ status. This may include determination of, e.g., ex vivo cellular cytokine release, phagocytosis activity, and/or antigen-presenting capacity. In this regard, standardized flow-cytometric assessment of the major histocompatibility-II complex human leukocyte antigen (-D related) (HLA-DR) has gained particular interest. Monocytic HLA-DR (mHLA-DR) controls the interplay between innate and adaptive immunity and may serve as a “global” biomarker of injury-associated immunosuppression, and its decreased expression is associated with adverse clinical outcomes (e.g., secondary infection risk, mortality). Importantly, recent data demonstrate that injury-associated immunosuppression can be reversed—opening up new therapeutic avenues in affected patients. Here we discuss the potential scientific and clinical value of assessment of functional immunity with a focus on monocytes/macrophages and review the current state of knowledge and potential perspectives for affected critically ill patients.

## Review

The immune system is an essential organ in higher life forms, and its dysfunction or “failure” may be life-threatening. In humans, the immune system is ubiquitously distributed within all organs and consists of humoral and cellular components organized in highly complex dynamic social network architecture-like structures [1]. Key functions of the immune system embrace injury control in inflammation/infection and tumor recognition/surveillance [1]. Despite its paramount importance, however, the immune system or “immune organ” is mostly overlooked on intensive care units (ICU) today [2–7]. This may at least partly be due to the fact that its functional status cannot be adequately assessed by use of routine biomarkers such as C-reactive protein, procalcitonin, or numerical distribution of leukocyte (sub)-sets. Nevertheless, numerical assessment of leukocyte (sub-)populations may provide important additional information, e.g., when considerably deranged [8–10].

**Fig. 1:**
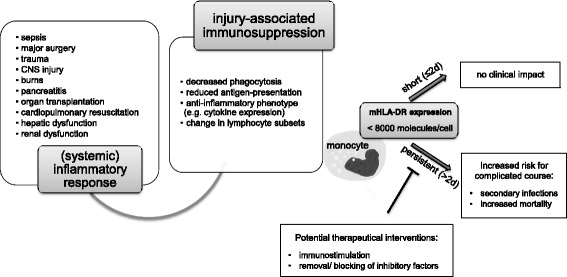
Injury-associated immunosuppression in critically ill patients. Injury-associated immunosuppression (IAI) may develop in critical illness. IAI was shown to be of importance in cases of persistence for ≥ 2 days. Key future potential therapeutical options are listed. Monocytic HLA-DR expression (mHLA-DR, given in bound antibodies per cell) may serve as a global marker of IAI

**Fig. 2:**
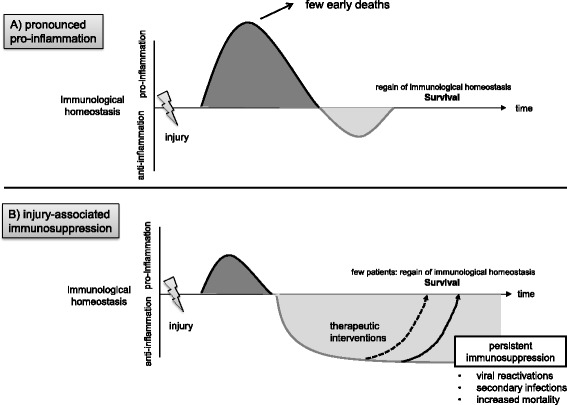
Inter-individual injury-associated response patterns in critically ill patients. Patients with critical illness respond differently to injury (e.g., sepsis). Whereas patient “A” undergoes a pronounced inflammatory phase (net effects are shown) with regain of immunological homeostasis and subsequent survival, patient “B” enters a persisting phase of injury-associated immunosuppression (IAI). In IAI, viral reactivation rates, secondary (re-) infection rates, and mortality is increased. This underlines the importance of inter-individual response patterns and need for individual patient characterization before application of interventional therapeutic approaches (adapted from Hotchkiss et al., 2013 [4])

**Fig. 3:**
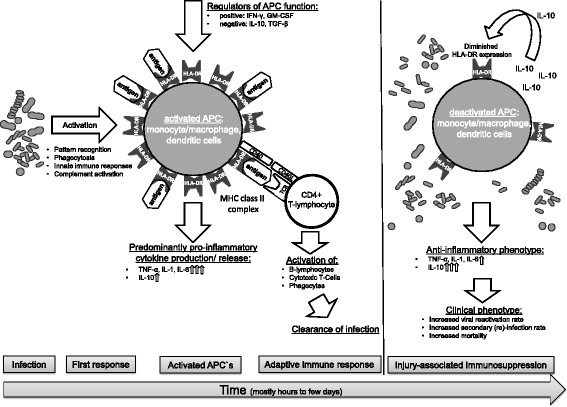
Infection-induced activation of key immune cells. In sepsis, bacterial infections trigger numerous pathways resulting in activation of key antigen-presenting cells (APCs) (i.e., monocytes/macrophages, dendritic cells). Activated APCs predominantly express pro-inflammatory cytokines and present antigens bound to major histocompatibility (MHC) class II complexes (such as HLA-DR). Antigen-bound HLA-DR triggers T-cell-receptor (TCR) and co-stimulatory molecule (e.g. CD 40-CD40L) binding. Adaptive immune responses are initiated resulting in clearance of infection. In, e.g., cases of overwhelming infection, deactivation of monocytes, as in sepsis-associated immunosuppression (SAI), may occur. SAI is characterized by a shift towards an anti-inflammatory phenotype with predominant expression of IL-10 and diminished HLA-DR expression, resulting in impaired clearance of infection and increased mortality. In IAI, the deactivated phenotype can be observed immediately after injury

The typical initial immune system response to critical illness consists of systemic and local release of inflammatory mediators and cytokines and activation of specific immune and other cells. This may lead to distinct phenotype changes in immune cells [4, 6, 11, 12]. The traditional understanding was that uncontrolled release of pro-inflammatory mediators (e.g., interleukin (IL)-1, tumor necrosis factor (TNF)-α) would determine adverse clinical outcomes in patients with septic shock [4, 11]. Consequently, anti-inflammatory such as anti-TNF-α or anti-lipopolysaccharide (LPS) strategies were then tested in large-scale clinical trials. However, respective trial results returned negative or indicated increased intervention-related mortality. This highlighted that an anti-inflammatory approach would not provide general benefits for larger populations of patients with sepsis/septic shock [2–5, 7, 12]. Thereafter, immune status characterization in larger patient cohorts using novel biomarkers allowed for a more profound understanding. When looking at an individuals’ immune response, a high inter-individual variance and highly dynamic changes can be observed over time (Figs. 1 and 2) [4]. Today, it is well established that many critically ill patients either show signs of co-existing inflammatory and counter-regulatory anti-inflammatory response early in critical illness [13, 14] or will undergo transition from early pro- to later anti-inflammatory phenotypes (Fig. 2) [2, 4, 7, 11, 12]. The “net effect” (i.e., the resulting phenotype) of such profound anti-inflammation was referred to as “sepsis- (or injury-) associated immunosuppression (SAI/IAI)” and embraces diminished release of pro-inflammatory mediators, reduced phagocytosis, and reduced expression of cellular surface receptors involved in antigen-presenting activity (e.g., major histocompatibility complex (MHC) class II) (Fig. 3) [4, 7, 11, 12]. This may be associated with enhanced immunological tolerance, increased immune cell apoptosis, and altered gene expression profiles [6, 11]. Interestingly, recent data show that respective changes are not exclusive to circulating immune cells and that comparable anti-inflammatory phenotypes can be found, e.g., in splenic or lung tissue and other solid organs [11].

From a clinical perspective, it seems pivotal to distinguish temporary from persisting immunosuppression (Figs. 2 and 3). Data show that patients failing to recover from injury- (or sepsis-) associated immunosuppression are at increased risk for (secondary) infections or non-survival [4, 6, 11, 15] (Fig. 2). This affects patients with post cardio-surgical conditions [16], trauma [17], burns [18], pancreatitis [19, 20], solid organ transplantation [21], hepatic [22] or renal injury [23], stroke [24], myocardial infarction/heart failure, and cardiac arrest [25–28], as well as sepsis [15]. Recent technological advances now allow for better recognition/monitoring of SAI/IAI—thus opening up new avenues for the recognition, monitoring, and treatment of such functional immune “organ failure” [7].

## Immunological markers in critical illness

For identification of patients at risk for SAI/IAI and associated complications, it seems important to briefly summarize key immunologic responses to injury (Fig. 3). The first response to injury or infection typically consists in local activation of humoral factors (e.g., complement factors) followed by antigen-presenting cells (APCs) that are at the innate-adaptive interface (i.e., monocytes/macrophages or dendritic cells) [6, 29]. When activated, APCs release cytokines (e.g., TNF-α, IL-1, IL-6) and other mediators that attract and activate even more APCs and neutrophils, enhance phagocytosis, and stimulate adaptive immune cells after migration to draining lymph nodes (e.g., antigen-loaded dendritic cells) [6, 29]. Following phagocytosis, APC-derived antigen presentation occurs via upregulation of class II transactivator (CIITA) and re-localization of MHC class II molecules from intracellular storages [29, 30]. Enhanced surface expression of antigen-loaded human leukocyte antigen (-D related) (HLA-DR; a key MHC class II molecule) on monocytes/macrophages and dendritic cells then induces a T cell response via binding to T cell receptors (TCR) and co-stimulatory molecules (e.g., CD86-CD28 and CD40-CD40L) (Fig. 3). Over time, a “counter-regulatory” response may occur in monocytes/macrophages and dendritic cells with increased production of anti-inflammatory cytokines (e.g., IL-10) [31, 32]. As a consequence, monocyte and dendritic cell deactivation with diminished expression of both HLA-DR and co-stimulatory molecules can be observed as an indicator of reduced phagocytosis, antigen presentation, and diminished induction of adaptive immune responses. Furthermore, expansion of myeloid-derived suppressor cells (MDSC), an immature population of myeloid cells with immunosuppressive functions first described in cancer, was also demonstrated in patients with sepsis [33, 34]. Very recently, MDSC were shown associated with prolonged immunosuppression, in particular with diminished T cell functions and development of nosocomial infections in patients with sepsis [35, 36]. In addition, critically ill patients commonly show marked apoptosis-induced lymphopenia and impaired lymphocyte function which contribute to sepsis- and injury-associated immunosuppression as recently reviewed elsewhere [37].

### Key cytokines: serum levels of IL-6, IL-10, and TNF-α

Serum cytokine levels are routinely assessed in some institutions for earlier recognition, estimation of prognosis, and (intra-individual) follow-up of critically ill patients. However, it should be noted that they do not reflect immune cell functionality as cytokines are mostly pleiotropic, derived from different cells including non-immune cells, may be counteracted by natural inhibitors (e.g., gp130 for IL-6), and have variable clearance rates [4, 6, 11, 12]. In the following, we discuss three cytokines with pathophysiologic and/or diagnostic relevance in critical illness:

IL-6 is a potent pleiotropic cytokine with mainly pro-inflammatory effector function. IL-6 is expressed by monocytes/macrophages, endothelial lineage cells, and fibroblasts and augments immune responses via induction of T cell activation, B cell proliferation and differentiation, and stimulates acute phase protein release (e.g. C-reactive protein) [38]. Systemic IL-6 is detected rapidly with peak serum levels observed after about 2 h after an inflammatory insult [38]. IL-6 is usually assessed via automated enzyme-linked immunosorbent assay (ELISA) in specialized laboratories or via point-of-care tests (blood, liquor) [39, 40]. Owing to its fast induction and short half-life, serial IL-6 assessment may provide timely monitoring of an inflammatory burden when, e.g., compared to serial C-reactive protein measurements. Although increased IL-6 levels indicate adverse clinical outcomes in adults with sepsis/septic shock [38], implementing of IL-6 measurement in routine diagnostic work up was not shown to improve patient-centered clinical outcomes. Nevertheless, IL-6 was shown useful for sepsis diagnostics in neonatal/pediatric critically ill patients [41].

IL-10 is regarded the most prominent and exemplary anti-inflammatory cytokine. Comparable to IL-6, IL-10 is mainly expressed by monocytes/macrophages, has a short half-life, and can be assessed by ELISA. IL-10 was evaluated in several studies and functionally linked to the “classical” biphasic response model to severe injury [42, 43]. In contrast to IL-6, increased IL-10 expression induces antigen tolerance, enhances SAI, and increases susceptibility to infection, and IL-10 blockade reverses endotoxin tolerance in several preclinical studies, and some reports show a predictive value of IL-10 for mortality and/or (secondary) infection [42, 43].

TNF-α is a key pro-inflammatory cytokine predominantly released by monocytes/macrophages in early sepsis. It auto-stimulates effector functions and enhances the initiation of adaptive immune responses [44]. Several studies showed that elevated TNF-α levels are associated with increased mortality. When compared to other systemic inflammatory markers, it appears that TNF-α has lower discriminatory power with respect to outcome prediction [43, 45].

### Functional markers: ex vivo TNF-α release

Ex vivo LPS-induced TNF-α production (e.g., after 4 h of stimulation) in whole blood allows for quantification of production/release of monocytes and dendritic cell-derived TNF-α. Diminished ex vivo TNF-α release is a key feature of immunosuppression in critically ill patients [4, 12, 46]. Nevertheless, ex vivo TNF-α release may not be a suitable diagnostic marker for cellular immune function as it requires standardized protocols for sample handling and specific stimulation conditions [46]. Today, no generally accepted standardized protocol for assessment of ex vivo TNF-α release exists, hindering multicenter studies [46]. Recently, whole-blood monocytic intracellular TNF-α assessment by flow cytometry was tested and showed promising results with regard to improved test feasibility [47].

### Functional markers: phagocytosis assays

Phagocytosis involves recognition and engulfment with subsequent clearance of pathogens [48]. Numerous predominantly innate immune cells perform phagocytosis (e.g., neutrophils, monocytes/macrophages, dendritic cells) [48]. Diminished phagocytic capability was linked to increased susceptibility for (secondary) infection in rodent models whereas in humans, the direct influence of critical illness on phagocytosis is incompletely understood [49]. Phagocytosis of neutrophils may be conserved in patients with sepsis, while in parallel, other neutrophil functions including chemotaxis and/or generation of oxidative burst may be impaired [49]. In general, phagocytosis assays are heterogeneous with varying specificity. Standardized laboratory protocols are missing, resulting in high intra- and inter-lab variation. Thus, phagocytosis assays may be of limited use for assessment of immune function in both clinical routine and multicenter clinical trials testing immunological interventions.

### Functional markers: mHLA-DR expression

HLA-DR is a MHC class II molecule and predominantly expressed on monocytes/macrophages, dendritic cells, and B cells [29]. Its surface expression is indispensable for antigen presentation [29]. While increased HLA-DR expression reflects activation of immune cells, diminished expression thereof exhibits a phenotype with downregulation of antigen-presenting capacity and a shift from pro- to anti-inflammatory cytokine production [4, 12]. Surface expression of HLA-DR on monocytes/macrophages is crucial for initiation of adaptive immune responses [11, 29]. This signal is paralleled and/or augmented by activation of co-stimulatory molecules (e.g., CD40- CD40-ligand binding) (Fig. 3). Given the importance of monocytic HLA-DR (mHLA-DR) expression in respect to induction of adaptive immune responses, the key interplay of monocytes and dendritic cells with T cells was colloquially referred to as “immunological synapsis.” Assessment of mHLA-DR expression was thus proposed to serve as a “global” functional marker of immune function [4, 5, 7, 12]. In fact, the significance of mHLA-DR expression was first described about 30 years ago in patients undergoing organ transplantation when patients with low HLA-DR expression could be weaned from iatrogenic immunosuppression without transplant rejection [50].

## Flow-cytometric assessment of mHLA-DR expression

**Fig. 4:**
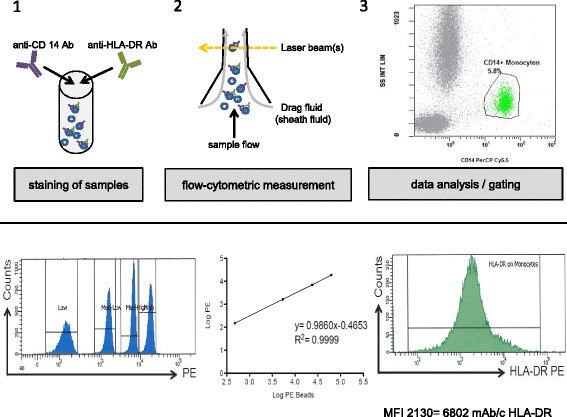
Flow-cytometric assessment of monocytic HLA-DR expression. Upper row: after staining of EDTA samples with specific antibodies, HLA-DR expression is assessed on CD14^+^ monocytes by flow cytometry. *Lower row*: (*left*) Quantibrite™-PE beads are used to calculate a calibration curve (middle) for HLA-DR assessment on CD14^+^ monocytes. (*Right*) mean fluorescence intensity (MFI) values for HLA-DR on monocytes are converted in a given sample to molecules per cell using the calibration curve

Monocytic HLA-DR expression is performed via fluorescence-activated cell sorting (FACS) from EDTA samples [51, 52]. FACS allows for simultaneous enumeration and assessment of several surface and intracellular antigens on specific immune cell subsets following staining with fluorochrome-labeled antibodies (Fig. 4). In 2005, the Quantibrite™ HLA-DR assay was demonstrated as the first standardized method for flow-cytometric mHLA-DR assessment with low inter-laboratory variability (coefficient of variation (CV) 15%, inter-laboratory CV < 4%) enabling comparison of data sets collected in multicenter studies [51]. Previous methods reporting percentages of HLA-DR positive cells (%HLA-DR) or mean fluorescence intensities (MFI) lacked an internationally accepted analytical standard and precluded between-center comparison of results [51]. In contrast, the Quantibrite™-HLA-DR assay harnesses calibration beads and a specifically formulated antibody-fluorochrome conjugate which allows the measurement of bound HLA-DR antibodies per cell (mAb/cell) independently from the combination of flow cytometer or instrument settings used in different laboratories [51]. Despite recent progress in standardization, flow cytometry still requires specialized lab equipment and staff, standardized analytical protocols, and timely handling of samples (maximum of 4–6 h in standard EDTA-tubes at room temperature for mHLA-DR) [51]. Delayed assessment of samples may induce activation of monocytes resulting in artificially increased mHLA-DR expression. Storage of EDTA-anticoagulated whole blood on ice or in a refrigerator or use of cell preservative containing tubes such as Cyto-Chex®-BCT increase analytic stability for mHLA-DR ([51] and Meisel et al., unpublished data). However, Cyto-Chex®-BCT tubes are expensive and not commonly available. Stained and fixed samples can be stored for at least 52 h before analysis [51]. Thus, mHLA-DR assessment as a biomarker for immune function usually requires establishing of the method in nearby hospital laboratories [7, 52]. In addition, blood samples are usually processed during standard laboratory opening hours and not 24/7 [51, 52]. Recently, an automated table cytometer was investigated as potential point-of-care tool for bedside mHLA-DR assessment which may be an important step to improve the availability of immune monitoring tools for ICU clinicians [53]. Further, quantification of HLA-DR expression and of other markers of innate and adaptive immune (dys)-regulation by real-time or digital PCR may help to overcome some of the above mentioned limitations of flow-cytometric mHLA-DR analysis and thus improve identification of patients with SAI/IAI [54–57]. However, the utility of theses assays needs further investigation.

### Threshold levels

Using the earlier non-standardized method for mHLA-DR assessment as percent positive monocytes, most investigators (including our group) have established a cut-off at 30% HLA-DR-positive monocytes for severe injury-associated immunosuppression (earlier referred to as “immunoparalysis”) [51]. A recent comparison of the conventional method with the standardized quantitative assay for mHLA-DR (given in mAb/cell) performed by us revealed that the (earlier) cut-off value of 30% HLA-DR positive monocytes corresponds to about 5000 mAb/cell and 45% mHLA-DR to about 8000 mAb/cell [51]. The range between 30 and 45% HLA-DR positive monocytes was termed “borderline immunosuppression.” Thus, a cut-off value of 8000 mAb/cell may be used to indicate SAI/IAI and was used in subsequent interventional clinical trials [58]. Importantly, not single diminished values of mHLA-DR should be regarded as clinically relevant but rather the persistence of low mHLA-DR levels indicating failure for recovery [4, 7, 12, 15].

## Monocytic HLA-DR expression in specific diseases

### Sepsis/septic shock

Sepsis is the clinical condition in which the mHLA-DR expression is best evaluated. Reduced mHLA-DR expression on admission [59, 60], days 1–3 [15, 45, 60] and days 6–8, [45, 59, 61] was significantly associated with increased mortality. Some studies show that the outcome-relevant difference in mHLA-DR expression is apparent only on days 3–4 (or later) with mHLA-DR returning to normal levels in survivors but not in non-survivors [15, 62]. Two further studies showed that the dynamic change (or recovery slope) in mHLA-DR expression between days 3 and 7 post injury is associated with mortality [15, 61, 62]. In one of these studies, it was shown that despite non-significant predictive value for single mHLA-DR values at time points 0, 3, and 7, the delta value between measurements days 0–3, 0–7, and 3–7 were highly predictive for mortality [62]. These results were confirmed in both adult [45] and pediatric patients [61]. One explanation for the better predictive value of relative changes in mHLA-DR expression than absolute values may be the high inter-individual variability of HLA-DR levels on monocytes. Monocytic HLA-DR expression on days 3–5 and 6–8 also independently predicts development of secondary infections [63]. Recovery of mHLA-DR may also reflect normalization of key metabolic pathways in sepsis [64–66], but further large-scale clinical data is needed.

### Major surgery

Several studies assessed whether reduced mHLA-DR expression predicts adverse outcome following major surgical procedures. Culprits for post-surgical immune suppression may be surgical trauma, related intraoperative hypotension [67], and increased perioperative release of corticosteroids or catecholamines [68]. Moreover, anesthetic drugs such as fentanyl [69] may contribute to injury-associated immunosuppression (IAI). In patients with cardiovascular surgery, use of extracorporeal circuits is typically associated with a substantial pro-inflammatory response [16]. Cardiopulmonary bypass may be followed by IAI reflected by impaired monocytic ex vivo LPS-induced cytokine release and decreased mHLA-DR [16, 70, 71]. The nadir of mHLA-DR was typically observed on postoperative days 1–3, but diminished mHLA-DR expression was shown to persist up to postoperative day 10 in a considerable number of patients [70, 71]. In two larger studies investigating the predictive power of mHLA-DR on outcome in pediatric and adult patients post-cardiac surgery, reduced mHLA-DR expression on postoperative day 3 was associated with increased length of ICU stay/mechanical ventilation and development of postoperative sepsis [71, 72] after adjustment for bypass time, cross clamp time, complexity of surgical procedure, and a pediatric mortality risk score [72]. In adults, mHLA-DR expression on postoperative days 1–5 was significantly different between patients who later developed sepsis vs. with an uncomplicated course and was a factor with a high discriminatory power to identify patients with infection post cardiac surgery [16]. In patients with ruptured abdominal aortic aneurysms, mHLA-DR expression after surgery was significantly associated with mortality although this was not related to increased postoperative infection rates [73].

### Multiple trauma

Diminished mHLA-DR expression was observed in many patients with multiple trauma [17, 74]. In a prospective observational trial in 105 severely injured patients (injury severity score, ISS > 25), rise in mHLA-DR until days 3–4 following trauma, and not at any earlier day, was associated with non-development of severe infection/sepsis after adjusting for confounders [17]. The dynamic effect of mHLA-DR recovery was also shown in patients with multiple trauma and ISS > 9 [75]. Further studies report an association between mHLA-DR expression and occurrence of post-trauma sepsis as early as day 2 [75]. Monocytic HLA-DR expression was also associated with increased intrapulmonary shunting after severe trauma which is associated with increased incidence of pulmonary sepsis and development of acute respiratory distress syndrome (ARDS) [75].

### Central nervous system (CNS) injury

Infection is a common complication in patients after acute CNS injury. In particular, pneumonia is associated with worse neurological outcome and remains a leading cause of death. Experimental studies demonstrate that CNS injury-induced suppression of cellular and humoral immune functions contribute to the high incidence of infections [24]. Several clinical studies demonstrated reduced mHLA-DR expression in patients after cerebral ischemia, subarachnoid hemorrhage, spinal cord injury, or neurosurgery [76–78]. Importantly, CNS-injured patients with subsequent infectious complications showed lower mHLA-DR levels than those with an uncomplicated clinical course as early as day 1 after the insult and well before onset of infection [76–78] indicating that impaired host responses contribute to an increased infection risk after CNS injury. Very recently, we confirmed in a large prospective multicenter study stroke-induced immunosuppression (as indicated by low mHLA-DR expression) as an independent risk factor for the development of pneumonia besides the known neurological risk factors leading, e.g., to dysphagia and higher risk of aspiration [77].

### Burn injury

Only few data are available in burn patients. One study in patients with severe burn injury (> 30% of body surface) indicates that days 2–3 mHLA-DR expression is significantly associated with increased mortality [18]. Patients who later developed sepsis had significantly lower mHLA-DR expression in the two ensuing days [18].

### Pancreatitis

Reduced mHLA-DR expression is associated with increased disease severity in patients with severe pancreatitis [19, 20]. Suppression of mHLA-DR or decreased mHLA-DR is associated with development of sepsis [19, 20]. After day 3, failure to recover in mHLA-DR expression was associated with decreased survival [19].

### Transplantation

The utility of mHLA-DR assessment in patients post (e.g., renal) transplantation was investigated more than 25 years ago. Increased mHLA-DR expression was observed to be associated with an increased rate of transplant rejection [21, 79] and may serve to monitor iatrogenic immunosuppression [50]. Failure to recover to normal mHLA-DR levels after transplantation is associated with increased rates of late post-transplant pneumonia in pediatric populations [80]. In adults after liver transplantation, reduced mHLA-DR expression levels are associated with pneumonia [81] and cytomegaly virus (CMV) reactivation [82].

### Cardiopulmonary arrest

Monocytic HLA-DR expression predicts outcome in patients after cardiac arrest (CA) [25]. In 55 patients after out-of-hospital CA from non-shockable rhythm, mHLA-DR levels were significantly decreased when compared to healthy controls [25]. In this study, non-survivors showed different mHLA-DR dynamics between days 0 to 1 and 1 to 3 when compared to survivors. Whereas the slope between days 0 and 1 was steeper in non-survivors, mHLA-DR expression continued to decrease from days 1 to 3 in non-survivors (increased after day 1 in survivors) [25].

### Other clinical conditions incl acute kidney injury and acute hepatic failure

The predictive value of mHLA-DR on outcome of patients with acute kidney injury (AKI) was assessed in one study [23]. Despite decreased mHLA-DR expression in AKI patients when compared to controls, the study did not identify a predictive value for mortality [23]. Few studies investigated mHLA-DR expression in patients with acute or decompensated chronic liver disease [22, 83]. Respective studies found a significant association between mHLA-DR expression and mortality at admission with an increase in predictive value when dynamic changes over time were investigated [83]. The discriminatory power of mHLA-DR for prediction of mortality was either similar [83] or lower than for the Model of End-Stage Liver Disease (MELD) score [22].

## Injury-associated immunosuppression: reversal by therapeutic interventions

**Fig. 5:**
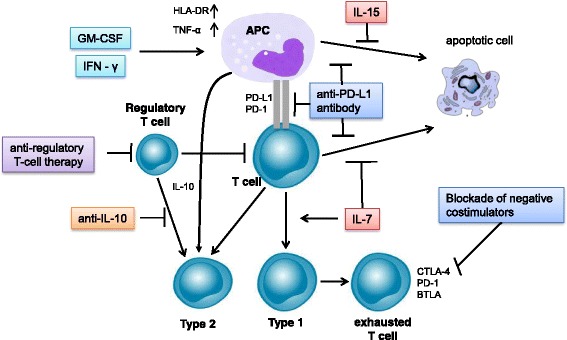
Potential future immunomodulatory approaches in sepsis. Key approaches to reverse sepsis-associated immunosuppression include cytokine-induced stimulation of monocyte/macrophage function (GM-CSF, IFN-γ), administration of survival factors for T cells (IL-7), blockade of anti-inflammatory mechanisms (anti-IL-10 antibody/antagonization of regulatory T-cell function), approaches to target immune cell exhaustion/apoptosis (anti-programmed death (PD) receptor 1 or PD-ligand1 (PD-L1)), and blockade of negative co-stimulators (e.g., cytotoxic T-lymphocyte-associated protein 4 [CTLA-4] or B- and T-lymphocyte attenuator (BTLA))

In the light of the potential of mHLA-DR for immune monitoring, several interventional biomarker-guided therapeutic strategies were tested in clinical trials. Respective approaches included extracorporeal removal of inhibiting factors via selective immunoadsorption [84], immunostimulation using interferon gamma (IFN-γ) [32] or stimulation with granulocyte-macrophage-colony-stimulating factor (GM-CSF) [58, 85, 86]. Potential additional approaches embrace interleukin 7 (IL-7) or anti-PD ligand 1 molecules (anti PD-L1). Future potential immunomodulatory approaches in sepsis are given in Fig. 5.

### Interferon gamma (IFN-γ)

Stimulation of IFN-γ receptors, which are ubiquitously expressed, results in activation of numerous pro-inflammatory pathways. In a landmark trial, Doecke et al. showed that IFN-γ immunostimulation restores mHLA-DR expression in patients with sepsis-associated immunosuppression (SAI) [32]. Clearance of infection may be enhanced by IFN-γ use in adult patients with invasive fungal sepsis [87], and in a randomized double-blind clinical trial in trauma, a decreased incidence for ventilator-associated pneumonia was observed in patients with mHLA-DR < 30% receiving inhaled IFN-γ [74]. IFN-γ treatment was shown to reverse SAI resulting in higher TNF-α-, decreased IL-10, and increased mHLA-DR levels indicating reversal of the SAI phenotype [44]. Whether administration of IFN-γ in IAI results in lower mortality of affected patients remains unclear and larger investigations are needed but, importantly, major side effects of IFN-γ-induced immunostimulation were not observed [32, 74].

### Granulocyte-macrophage-colony-stimulating factor (GM-CSF)

In a randomized controlled double-blind placebo-controlled trial in 38 patients with sepsis, we could demonstrate reversal of persisting SAI following one treatment of subcutaneous GM-CSF [58]. In addition to reversal of SAI (as defined by mHLA-DR expression > 15,000 mAb/cell), we observed improvements in relevant patient-centered outcomes such as shortened time of mechanical ventilation [58]. The finding that GM-CSF reverses SAI is supported by other groups [86]. Whether clinical endpoints such as secondary infection rates are affected by therapeutical application of GM-CSF is under research (NCT02361528).However, smaller studies showed promising results with lower infection rates [88] or shorter duration of infection in immunosuppressed critically ill patients treated with GM-CSF. In another randomized-controlled trial in patients with sepsis and severe respiratory dysfunction, oxygenation significantly improved in patients receiving GM-CSF [89]. In newborns, we could recently demonstrate that reduced mHLA-DR expression may reflect immunological immaturity in very early newborns [90] and a meta-analysis on GM-CSF therapy indicated increased survival rates in very-low pre-term infants (< 2000 g) and infants with neutropenia when treated with GM-CSF [91]. Importantly, none of the clinical studies reported relevant side effects of GM-CSF treatment.

## Conclusions

Critical illness may often induce persisting injury-associated immunosuppression with adverse effects on relevant patient-centered outcomes. However, despite the key task of ICU physicians to detect, monitor, and follow up on organ dysfunctions, functional failure of the “immune organ” seems currently mostly overlooked as it cannot be adequately assessed via use of routine biomarkers such as numerical distribution of leukocyte (sub)counts or systemic levels of soluble markers such as cytokines, procalcitonin, or acute phase proteins. Importantly, quantitative assessment of a given cell population does not per se allow to conclude on its functional status.

Today, flow-cytometric assessment of the mHLA-DR expression may serve as a standardized “global” biomarker to evaluate immune *function*. Persisting reduced mHLA-DR expression reflects a distinct immunological phenotype that is associated with adverse clinical outcomes. Nevertheless, mHLA-DR assessment currently requires specialized laboratories that may not be available in all institutions. Following demonstration of immunological efficiency, biomarker-guided immunological interventions for injury-associated immunosuppression should now be performed in adequately characterized populations using relevant patient-centered clinical outcomes (e.g., mortality). We postulate that in the future of intensive care, personalized medicine that considers the individual immune functionality will be needed to significantly improve the outcome of affected patients.

## Abbreviations

APC: Antigen-presenting cell
CD: Cluster of differentiation
CIITA: Class II transcriptor activator
CMV: Cytomegaly virus
CNS: Central nervous system
CV: Coefficient of variation
EDTA: Ethylene-diamin-tetra-acetat
ELISA: Enzyme-linked immunosorbent assay
HLA(-DR): Human leukocyte antigen (-D related)
IAI: Injury-associated immunosuppression
IFN(-γ): Interferon (gamma)
IL: Interleukin
LPS: Lipopolysaccharide
MHC: Major histocompatibility complex
mHLA-DR: Monocytic HLA-DR
PCR: Polymerase chain reaction
PD: Programmed death
SAI: Sepsis-associated immunosuppression
TNF (-α): Tumor necrosis factor (alpha)
